# Genotype-dependent associations between serotonin transporter gene (*SLC6A4*) DNA methylation and late-life depression

**DOI:** 10.1186/s12888-018-1850-4

**Published:** 2018-09-04

**Authors:** Dilys Lam, Marie-Laure Ancelin, Karen Ritchie, Rosanne Freak-Poli, Richard Saffery, Joanne Ryan

**Affiliations:** 1 Cancer & Disease Epigenetics, Murdoch Children’s Research Institute, Royal Children’s Hospital, Parkville, VIC Australia; 2 Department of Paediatrics, University of Melbourne, Parkville, VIC Australia; 3 INSERM, Univ Montpellier, Neuropsychiatry: Epidemiological and Clinical Research, Montpellier, France; 4 Centre for Clinical Brain Sciences, University of Edinburgh, Edinburgh, UK; 5 Department of Epidemiology and Preventive Medicine, Monash University, Level 5, The Alfred Centre, 99 Commercial Road, Melbourne, 3004 Australia

**Keywords:** Depression, DNA methylation, Epigenetics, Genetic variation, Serotonin transporter

## Abstract

**Background:**

Disrupted serotonergic signaling is often a feature of depression and the role of the serotonin transporter gene (*SLC6A4*), responsible for serotonin re-uptake, has received much attention in this regard. Most studies have focused on the polymorphic *5-HTTLPR* upstream repeat, or DNA methylation at the promoter CpG island. Few studies have explored the influence of genetic variation across the gene on DNA methylation, and their combined association with depression risk. The aim of this study was to determine whether genetic variation in the *SLC6A4* gene influences promoter DNA methylation, and whether these are associated with depression status.

**Method:**

The ESPRIT study involves a community-based population of older individuals (> 65 years of age). Major depressive disorder (MDD) was diagnosed according to DSM-IV (American Psychiatric Association, 1994) criteria, and severe depressive symptoms assessed by the Centre for Epidemiological Studies Depression (CES-D) Scale. Sequenom MassARRAY was used to measure *SLC6A4* methylation status (*n* = 302).

**Results:**

Nominally significant associations were observed between *SLC6A4* genetic variants (5*-HTTLPR*, *rs140700, rs4251417, rs6354, rs25528, rs25531)* and DNA methylation at several CpG sites. In multivariate regression, DNA methylation was associated with depression status, but only in the presence of specific genotypes. In individuals homozygous for the short *5-HTTLPR* and *5-HTTLPR/r25531* alleles, lower methylation at two CpGs was associated with depression (*β* = − 0.44 to *β* = − 0.31; *p* = 0.001 to *p* = 0.038).

**Conclusion:**

We present evidence for genotype-dependent associations between *SLC6A4* methylation and depression. Genetic variants may also play a role in influencing promoter methylation levels and its association with depression.

**Electronic supplementary material:**

The online version of this article (10.1186/s12888-018-1850-4) contains supplementary material, which is available to authorized users.

## Background

Serotonin deficiency was one of the earliest hypothesis of depression causality [1, 2]. This came from the discovery of a class of antidepressants, the selective serotonin re-uptake inhibitors (SSRIs), which block the reuptake of the neurotransmitter and therefore increasing its availability. SSRIs are thus commonly prescribed to treat depression, however the response rate remains relatively modest (around 50%) [3]. The serotonin transporter (5-HTT), encoded by the serotonin transporter gene (*SLC6A4*), is a principle regulator of serotonergic neurotransmission and the active target of SSRIs [4]. Thus, the role of *SLC6A4* and its (dys)regulation has been a major focus of depression research.

One feature of *SLC6A4* which has been the subject of much research, is the upstream gene-linked polymorphic region, *5-HTTLPR*. A 44 bp insertion/deletion variable number tandem repeat (VNTR), results in either short (S) or long (L) alleles. The S allele results in lower *SLC6A4* transcriptional levels and therefore reduced reuptake of serotonin [5, 6]. There is some evidence that *5-HTTLPR* genotype affects vulnerability to a broad range of behavioural disorders including depression [1]. However, these associations have not been consistently found [7], suggesting that other regulatory mechanisms and factors are likely to contribute to depression risk [8–10].

Approximately 1 kb downstream from *5-HTTLPR* lies another widely studied regulatory element, a CpG island that spans the upstream promoter of exon 1 and the transcriptional start site [11]. DNA methylation of this compact region of CpG sites has been associated with reduced expression [11]. Previous, generally small studies have investigated the association between depression and *SLC6A4* DNA methylation in peripheral tissues, but with conflicting findings [12–20]. It is now clear that genetic variation is an important regulator of DNA methylation across the genome [21], yet only a few studies have examined whether *5-HTTLPR* influences methylation and/or the association between methylation and depression [15, 16]. To date the effects of other *SLC6A4* genetic variants on DNA methylation and on the potential link between depression and methylation, have yet to be investigated.

Using data gathered as part of a large general population cohort study of older persons, this study firstly investigated whether *SLC6A4* genetic variation across the gene influences promoter DNA methylation and secondly, whether depression is associated with DNA methylation independently and/or in combination with genetic variation.

## Methods

### The ESPRIT study

Participants included in this study were part of the French ESPRIT study of neuropsychiatric disorders in an older population [22]. Eligible participants aged 65 years and older from the non-institutionalised general population were randomly recruited from electoral rolls within the Montpellier district. Recruited participants to ESPRIT provided written informed consent and were asked to undergo standardized health and psychiatric interviews, as well as extensive clinical assessments. Information was collected on the participants’ lifestyle, health and medical use. The study protocol was approved by the Ethical Committee of University Hospital of Kremlin-Bicêtre, France.

### Depression measures

Major depressive disorder (MDD) was diagnosed according to the Diagnostic and Statistical Manual of Mental Disorders-IV (DSM-IV) (American Psychiatric Association, 1994) criteria, using the Mini International Neuropsychiatric Interview (MINI, French version 5.00) [23]. Diagnoses were further reviewed and validated by a panel of psychiatrists and a psychologist with access to information from participants’ health assessments. Severity of depressive symptoms was assessed by the Centre for Epidemiological Studies-Depression (CES-D) scale, a self-reporting questionnaire previously validated within the older population [24, 25]. A score of 16 or above is considered the threshold of depressive symptoms warranting further clinical investigation [25]. Thus, late-life depression was defined in this study as having a diagnosis of MDD or high levels of depressive symptoms (CES-D ≥ 16).

### Genotyping

Blood samples were collected at recruitment following clinical assessment. In concordance with previous *SLC6A4* methylation association studies in blood [12–15, 17, 19, 20], genomic DNA was extracted from white blood cells using a standard procedure [26] and used for genotyping and methylation analysis. Along with *5-HTTLPR*, five single nucleotide polymorphisms (SNPs) (*rs140700, rs25528, rs4251417, rs6354, rs25531*) spanning the *SLC6A4* gene were also genotyped. These were chosen on the basis of allele frequencies and prior associations with depression [3, 26, 27]. Genotyping of *5-HTTLPR* was performed as previously described [26], and SNPs were genotyped by KBiosciences (UK), using the KBioscience Competitive Allele-Specific PCR SNP genotyping system (KASPar) [28]. *5-HTTLPR* can also be considered in combination with *rs25531*, a SNP that lies within the repeat region and has been reported to modify transcriptional activity of *5-HTTLPR* [27, 29]. *5-HTTLPR/rs25531* describes the triallelic polymorphism accounting for both *5-HTTLPR* and *rs25531* genotypes. χ^2^-tests were used to calculate whether the distribution of genotype frequencies was in Hardy-Weinberg equilibrium (HWE).

### SLC6A4 promoter methylation analysis

500 ng of genomic DNA was sodium bisulphite-converted using the EZ-96 DNA Methylation-Lightning MagPrep kit protocol (Zymo Research; Irvine, USA). The promoter-associated CpG island was targeted in our assay. This region is equivalent to amplicon 1 in a previous study [30]. A 335 bp region (UCSC Human Genome Browser GRCh/hg_38 build: chr17: 30235734–30,236,068) [31] was amplified in triplicate, to account for variation in the PCR step [32], and methylation of 11 CpG units, encompassing 20 CpG sites, were quantified using the SEQUENOM MassARRAY EpiTYPER platform (Additional file 1: Table S1) [32]. Raw methylation data was generated on 361 samples. Mean methylation values were calculated from replicates within 10% of the median value (Martino et al. 2013). Participants with < 50% of methylation data available (*n* = 55) and outliers (> 3 times the interquartile range (IQR)) (*n* = 4) were excluded from further analysis. Following these quality control steps (Additional file 1: Figure S1), methylation data was obtained for a sub-sample of 302 participants, with no significant difference in depression status, age, sex and key variables (Table 1) with the full ESPRIT cohort (*p* > 0.05 for all comparisons).

**Table 1:** Characteristics of study participants according to depression status

Characteristic^a^	No depression	Depression	*p*- value^g^
*n*	207	95	–
Age (Mean ± S.D.)	72.5 ± 5.2	74.6 ± 5.7	0.004
	Proportion (%)		
Female	55.1	68.4	0.028
Past major depressive disorder	25.3	38.6	0.062
High education level^b^	38.2	25.3	0.028
Living alone	20.9	43.2	< 0.001
Habitual alcohol drinkers (> 24 g/day)	19.2	20.4	0.81
Habitual smokers (≥10 pack years)	35.6	34.4	0.84
Functional impairment^c^	5.31	13.7	0.013
Ischemic disease^d^	10.6	22.1	0.008
Comorbidities^e^	12.1	29.5	< 0.001
Anxiety	15.9	29.5	0.044
Dementia	4.3	2.1	0.334
Impaired cognition (MMSE< 26)^f^	13.6	11.6	0.63
Antidepressants	3.38	12.6	0.002
Selective serotonin re-uptake inhibitor (SSRI)	2.90	6.3	0.158
Tricyclic antidepressant (TCA)	0.48	2.10	0.187
Other antidepressants	0.00	4.20	0.003
Benzodiazepines	4.83	9.47	0.123
Anxiolytics	7.73	16.8	0.017
Psychotropic drugs	4.83	11.6	0.032
Anticholinergics	4.83	7.37	0.374

^a^Not all participants with methylation data (n = 302) had information for each population characteristic listed, but missing data was < 2%, with exception of anxiety (20.5%) and past MDD (33%)

^b^Underwent post-secondary education of any type

^c^Unable to independently complete 2 items on both or either of the Instrumental Activities of Daily Living and Activities of Daily Living scales

^d^History of angina pectoris, myocardial infarction, stroke, cardiovascular surgery and/or arteritis

^e^Hypertension (resting blood pressure ≥ 160/95 mmHg), high cholesterol (total cholesterol ≥6.2 mmol/l), diabetes (fasting glucose ≥7.0 mmol/l), thyroid disease, asthma, or recent cancer diagnosed within the last 2 years

^f^MMSE: Mini-Mental State Examination

^g^Chi-squared tests used to assess *p*-value for all variables except age, where a t-test was used

### Statistical analysis

Statistical analyses were performed using the statistical software package Stata 14.1 (StataCorp, College Station, Texas, USA). Univariate analysis (analysis of variance, t-tests, χ^2^-tests) was performed to examine potential associations between genotype and DNA methylation, and between depression and DNA methylation. Additionally, these statistical tests were used to determine which population characteristics were associated with depression status (Table 1)and/or DNA methylation levels independently. This step was performed to identify potential confounding factors of the association between DNA methylation and depression, which thus would be considered further in multivariate analysis. For associations between population characteristics and methylation, the significance threshold was set at a conservative level of *p* < 0.15, to ensure that no potential confounding variables were omitted. Characteristics associated with both depression and methylation were considered in subsequent multivariate regression analysis as covariates. This included age, sex, living alone, functional impairment, ischemic disease, anxiety, comorbidities. Multivariate linear regression models were used to model the association between DNA methylation (outcome variable) and depression (predictor variable), while adjusting for the potential confounding factors. Potential modifying effects of genetic variants on the association between depression and DNA methylation were also investigated through the inclusion of a multiplicative interaction term in the multivariate models. When potential modifying effects of a specific genetic variant were found (at *p* < 0.15), stratified analysis of methylation data across the genotype groups was performed. This involved t-tests to determine the association between depression status and DNA methylation, in each genotype group of the specific variant. Sensitivity analysis excluding antidepressant users (*n* = 19) was performed as antidepressants may potentially mask depression status and independently influence methylation levels [33]. Sensitivity analysis excluding participants with past depression (*n* = 59) was also performed. Correction for multiple testing was performed using the Benjamini-Hochberg false discovery rate (FDR) method (FDR = 0.05).

## Results

### Study population

In this study of 302 participants, depressed individuals (31.5%) were significantly more likely to be female, of older age, have a lower education level, live alone, be functionally impaired and have poorer health (ischemic pathologies and comorbidities), as compared to non-depressed participants. They were also more likely to be taking antidepressants. Characteristics of participants according to depression are shown in Table 1. All genotypes were in HWE (*p* > 0.05 for all polymorphisms). Of the six genetic variants and one combined variant examined, only *5-HTTLPR* was significantly associated with depression (Additional file 1: Table S2).

### Association between promoter DNA methylation and SLC6A4 genetic variants

**Fig. 1:**
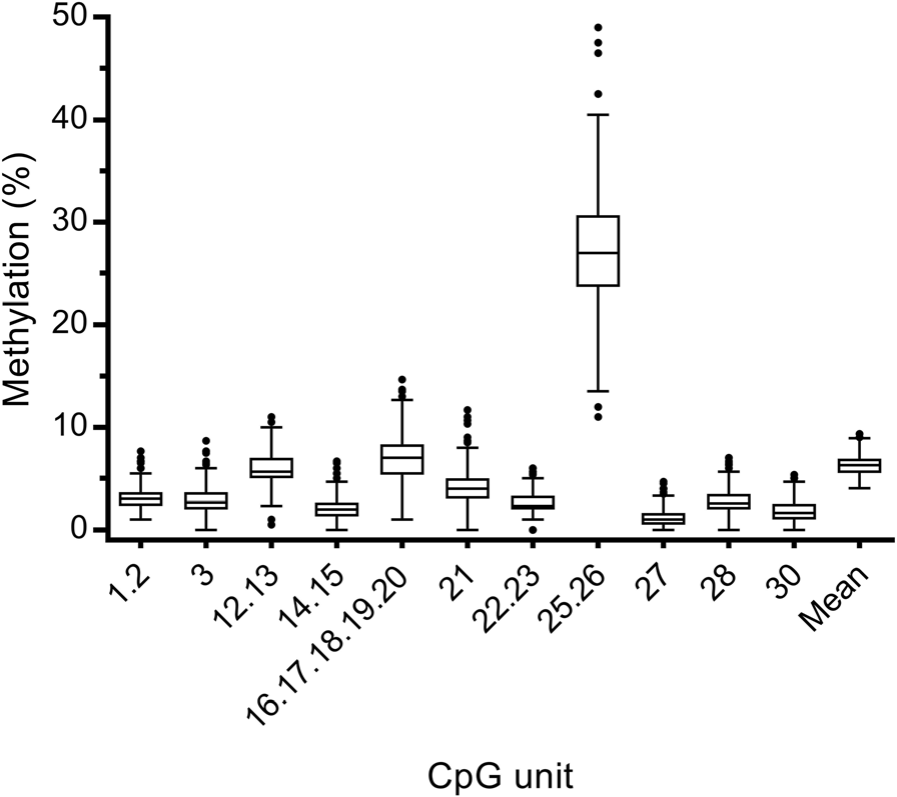
Distribution of *SLC6A4* methylation levels at each CpG unit and mean methylation levels across the region studied for all study participants. The box denotes the interquartile range (IQR: 25–75% percentiles), the line shows the median and the whiskers are 1.5 x IQR below the 25% percentile or above the 75% percentile

Overall, the methylation levels at the 335 bp promoter region were relatively low, apart from CpG 25.26 which had the highest and most variable distribution of methylation (Fig. 1). Potential associations were observed between specific genetic variants and DNA methylation, independent of depression status (Table 2). In particular, homozygous GG genotypes of *rs140700* (*p* = 0.019) and *rs25531* (*p* = 0.007) were significantly associated with higher methylation at CpG 16–20, while homozygous CC genotypes of *rs25528* (*p* = 0.007) and *rs6354* (*p* = 0.023) had significantly higher methylation at CpG 21. However, it should be noted that applying a FDR Benjamini-Hochberg correction for multiple testing of 11 CpG units and 6 polymorphisms (FDR = 0.05) abolished these associations.

**Table 2:** Potential associations between *SLC6A4* DNA methylation and genotype

CpG unit	Genetic variant	Average methylation (%)			*p*-value^a^
		Major homozygote	Heterozygote	Minor homozygote	
3	*rs4251417*	GG (3.04)	GA (2.70)	AA (1.56)	0.12
14.15	*rs4251417*	GG (2.26)	GA (1.72)	AA (1.67)	0.095
16.17.18.19.20	*rs140700*	GG (7.14)	GA (5.73)	AA (4.92)	0.019
	*rs6354*	AA (7.29)	AC (6.43)	CC (6.03)	0.054
	*rs25531*	AA (7.06)	AG (5.74)	GG (8.44)	0.007
21	*rs25528*	AA (3.54)	AC (4.55)	CC (4.94)	0.007
	*rs4251417*	GG (4.03)	GA (3.17)	AA (3.06)	0.085
	*rs6354*	AA (3.53)	AC (4.30)	CC (4.94)	0.023
	*5-HTTLPR*	LL (3.85)	SL (4.03)	SS (4.55)	0.077

^a^*p*-values calculated by one-way ANOVAs and only those with *p* < 0.15 shown

### Association between depression and methylation

In unadjusted analysis, no significant differences in methylation were noted between depressed and non-depressed participants at any individual CpG units or the mean methylation across the region (*p* > 0.15, except for CpG 3 with effect size ∆ = 0.31%; 95% CI: 0.04; 0.67%, *p* = 0.084 comparing depressed with non-depressed individuals). These findings did not change after inclusion of potential confounders in regression models (as stated in methods), or in sensitivity analysis excluding antidepressant users (*n* = 19) or participants with past depression (*n* = 59).

### Modification of the association between depression and methylation by SLC6A4 genotype

Five *SLC6A4* polymorphisms were found to potentially modify the association between depression and DNA methylation at multiple CpG units. *5-HTTLPR* and *5-HTTLPR/rs25531* polymorphisms significantly modified the depression-methylation associations at both CpG 21 (*p*-values for interaction term: *5-HTTLPR, p* = 0.001; *5-HTTLPR/rs25531, p* = 0.006) and CpG 25.26 (*5-HTTLPR, p* = 0.064; *5-HTTLPR/rs25531*, *p* = 0.030). In light of this, analysis was stratified by genotype, where clear differences in associations were observed.

**Fig. 2:**
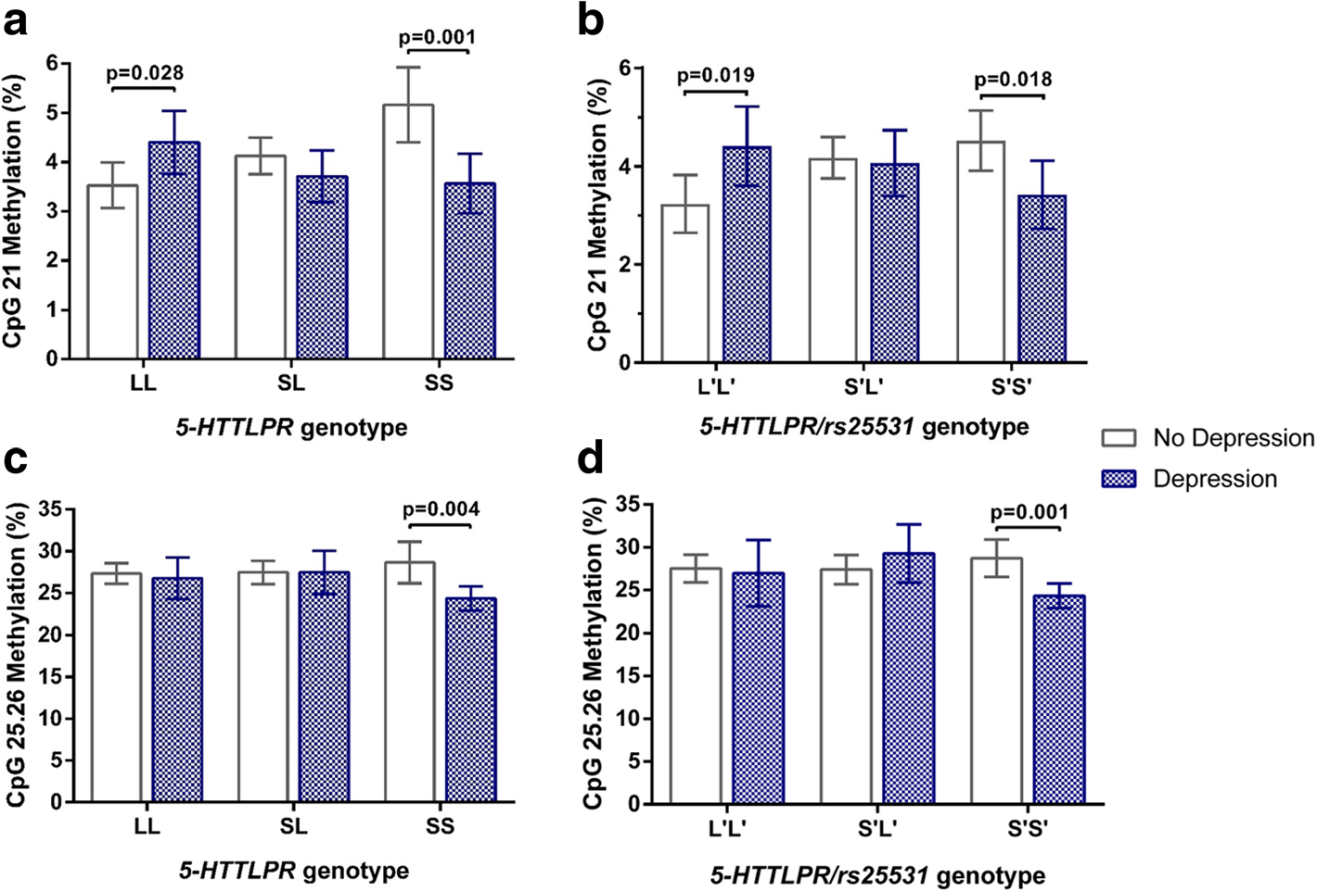
DNA methylation at CpG units 21 and 25.26 according to depression status, stratified by serotonin transporter (*SLC6A4*) genotypes: *5-HTTLPR* and *5-HTTLPR/rs25531.* **a** CpG 21, *5-HTTLPR* (*n* = 274); (**b**) CpG 21, *5-HTTLPR/rs25531* (*n* = 210); (**c**) CpG 25.26, *5-HTTLPR* (*n* = 234); (**d**) CpG 25.26, *5-HTTLPR/rs25531* (*n* = 182). Data presented as mean methylation ±95% CI. T-tests were used to calculate *p*-values, with significant associations at *p* < 0.05 indicated in the figure

Depression was significantly associated with lower methylation levels at CpG 21 and CpG 25.26, but only for individuals with the SS genotype of *5-HTTLPR* (SS, ∆ = − 1.60%; 95% CI: -2.54; − 0.65%; *p* = 0.001, Fig. 2a and ∆ = − 4.31%; 95% CI: -7.14; − 1.48%; *p* = 0.004, Fig. 2c respectively**).** In contrast, individuals with the LL genotype had highermethylation at CpG 21 (LL, ∆ = 0.88%; 95% CI: 0.10; 1.65%; *p* = 0.028, Fig. 2a). Similar genotype dependent associations were likewise observed for *5-HTTLPR/rs25531* at CpG 21 (S’S′, ∆ = − 1.10%; 95% CI: -2.01; − 0.20; *p* = 0.018, Fig. 2b) and CpG 25.26 (S’S′, ∆ = − 4.39%; 95% CI: -6.95; − 1.84%; *p* = 0.001, Fig. 2d**)** and at CpG 21 for L’L’ genotype (∆ = 1.18%; 95% CI: 0.21; 2.15; *p* = 0.019, Fig. 2b). These associations remained significant following multivariate adjustment for potential confounders, as shown in Table 3. Following correction for multiple testing (FDR = 0.05), associations between depression and methylation at CpGs 21 (*5-HTTLPR,* SS) and 25.26 (*5HTTLPR,* SS; *5HTTLPR/rs25531*, S’S′) remained significant.

**Table 3:** Adjusted associations between depression and *SLC6A4* promoter methylation, stratified by 5-*HTTLPR* or *5-HTTLPR*/*rs25531* genotype

CpG unit	Genotype	Variables	Unadjusted		Adjusted^a^	
			β(SE)	*p*-value	β(SE)	*p*-value
21	*5-HTTLPR* (LL)	Depression	0.24 (0.39)	0.026	0.28 (0.41)	0.016
		Female sex			− 0.092 (0.39)	0.39
		Age (yrs)			0.024 (0.036)	0.83
		Antidepressants			−0.15 (0.83)	0.18
	*5-HTTLPR* (SS)	Depression	−0.38 (0.52)	0.004	−0.44 (0.54)	0.001
		Female sex			−0.17 (0.58)	0.22
		Age (yrs)			−0.03 (0.068)	0.82
		Antidepressant			0.13 (0.96)	0.33
		Living alone			0.38 (0.69)	0.018
	*5-HTTLPR/rs25531* (L’L)	Depression	0.33 (0.49)	0.019	0.36 (0.53)	0.019
		Female sex			0.074 (0.48)	0.60
		Age (yrs)			−0.005 (0.057)	0.97
		Antidepressant			−0.093 (1.3)	0.54
	*5-HTTLPR/rs25531* (S’S′)	Depression	−0.28 (0.49)	0.027	−0.30 (0.50)	0.023
		Female sex			−0.14 (0.49)	0.29
		Age (yrs)			−0.22 (0.59)	0.097
		Antidepressant			0.098 (0.87)	0.44
		Living alone			0.27 (0.60)	0.056
25.26	*5-HTTLPR* (SS)	Depression	−0.37 (1.58)	0.009	−0.31 (1.68)	0.038
		Sex			−0.26 (1.65)	0.068
		Age (yrs)			−0.076 (0.20)	0.60
		Antidepressant			−0.087 (3.06)	0.56
	*5-HTTLPR/rs25531* (S’S′)	Depression	−0.37 (1.53)	0.006	−0.31 (1.52)	0.020
		Sex			−0.19 (1.46)	0.13
		Age (yrs)			−0.26 (0.18)	0.046
		Antidepressant			−0.11 (2.71)	0.39

^a^Adjusted for age, sex and antidepressant use, plus confounding factors (see methods) which remained significant in the final models at *p* < 0.15

Three other genotypes were also found to potentially modify the associations between depression and methylation: *rs140700* (mean methylation; *p*-values for interaction term = 0.083), *rs6354* (CpG 25.26; *p* = 0.061) and *rs4251417* (CpG 27, *p* = 0.026). Following stratification; mean methylation was significantly lower in depressed patients with heterozygote *rs140700* genotype (GA, ∆ = − 1.17%; 95% CI: -2.11; − 0.24%; *p* = 0.021; Additional file 1: Figure S2A); CpG 25.26 had highermethylation in depression for heterozygote *rs6354* genotype (AC, ∆ = 4.22%; 95% CI: 0.13; 8.32%; *p* = 0.044; Additional file 1: Figure S2B) and CpG 27 exhibited lowermethylation in depressed participants with homozygote *rs4251417* genotype (GG, ∆ = − 0.40%; 95% CI: -0.74; − 0.062%; *p* = 0.021; Additional file 1: Figure S2C). However, none of these associations remained significant in multivariate linear regression models (data not shown).

**Fig. 3:**
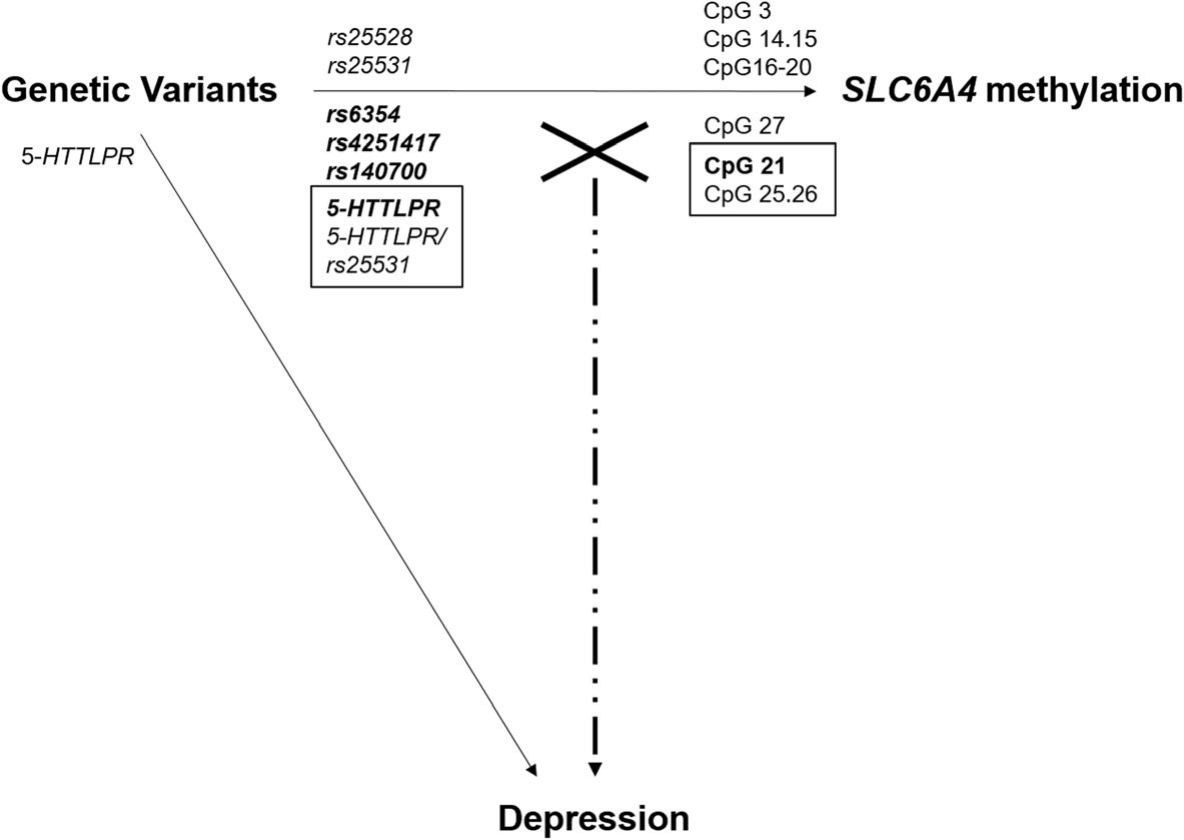
Diagram of relationships between *SLC6A4* genetic variants, promoter methylation and depression. Solid lines indicate an association. The X denotes the interaction between genetic variants and *SLC6A4* methylation in association with depression (dot-dash line). Bolded genetic variants or DNA methylation sites in boxes were independently associated, but also interacted to influence depression risk. Interactions which remained significant following FDR correction are boxed

The overall relationships between *SLC6A4* genetic variants, promoter methylation and depression are shown in Fig. 3.

## Discussion

Our study investigated the relationship between *SLC6A4* promoter methylation and six polymorphisms (*5-HTTLPR, rs140700, rs42151417, rs6354, rs25528, rs25531*), plus a combined variant (*5-HTTLPR/rs25531*) independently and in the context of late-life depression. We found marginal and nominally significant evidence that six genetic variants influenced site-specific methylation at four out of 11 CpG units. Whilst methylation was not independently associated with depression, specific genotypes were found to modify the association between depression and methylation at several CpG units. In particular, for individuals with the SS or S’S genotypes of *5-HTTLPR* and *5-HTTLPR/rs25531* respectively, depression was significantly associated with lowermethylation at CpG 21 and CpG 25.26. On the other hand, for individuals carrying the LL or L’L’ genotypes, depression was nominally associated with higher methylation at CpG 21. These findings were not confounded by sex, age, antidepressant use, or other factors examined including living situation and comorbidities.

Differential *SLC6A4* methylation at the promoter CpG island has been correlated with risk factors and adversities related to depression. Associations has been observed between maternal depression and decreased infant *SLC6A4* promoter methylation [34]. In adults, increased methylation has been associated with childhood trauma [35] and stress (early life and recent) [30, 36]. An inverse correlation between *SLC6A4* mRNA levels and promoter methylation has been demonstrated [11]. Several studies support our findings, reporting no association between *SLC6A4* methylation in blood and depression in adults, in either Caucasian [12] or three Asian (*n* = 108; *n* = 286; *n* = 100) [14, 15, 17] populations, as well as in buccal mucosa from Caucasian adolescents (*n* = 150) [16] and EBV-transformed lymphoblasts (*n* = 192) [18]. In contrast, three studies found positive associations between peripheral *SLC6A4* methylation in blood and depression in Caucasian (*n* = 57) [13] and Asian adults (*n* = 151; *n* = 84) [19, 20], two of which had overlapping assay regions with our study [19, 20]. Interestingly, Shi et al. (2016) found significantly highermethylation at two CpG sites (∆ = 2.52 and 0.15) corresponding to CpG 25.26 in our study, which we failed to find, despite this unit having the highest level and variability of methylation.

It is increasingly clear that genetic variation plays a critical role in the regulation of DNA methylation [21]. Such loci, referred to as methylation quantitative trait loci (mQTLs), may also modify the association between DNA methylation risk of disease [37, 38]. Previous studies that have investigated the extent to which *SLC6A4* genotype can influence DNA methylation in the context of depression, have focused primarily on the *5-HTTLPR* polymorphism. Several reported no significant association with mean methylation [12, 16, 17], which is supported by our findings. However, we did observe a trend for an association between *5-HTTLPR* SS genotype and higherCpG 21 methylation (*p* = 0.077), which is in concordance with a study in post-stroke depression [15]. In addition, *rs25531*, which has been shown to modify the functionality of *5-HTTLPR* [27, 29] was also associated with site-specific methylation at CpG unit 16–20. Here we have shown for the first time that four other polymorphisms (*rs4251417, rs140700, rs6354* and *rs25528*) in *SLC6A4* may potentially regulate DNA methylation in the region.

Modifying effects of *5-HTTLPR* genotype on the association between *SLC6A4* methylation and several depression-related adversities, including childhood abuse [12] and stress [36], have been demonstrated previously. The few studies which have investigated this in the context of depression, have found that the S allele in combination with higher*SLC6A4* methylation increased the risk of depressive symptoms in adolescents [16] and adults following stroke [15]. This contrasts with our current study which observed lower methylation at CpG units 21 and 25.26 in depressed older individuals with the SS genotype. However, a recent study by Iga et al. (2016) (average age: 42.2–45.0 years) found that the L allele was associated with higherperipheral blood methylation in depressed patients [13]. More broadly, as an inverse correlation exists between *SLC6A4* methylation and mRNA levels [11], our finding of decreased methylation in depressed individuals is consistent with the observation of increased peripheral *SLC6A4* mRNA in un-medicated MDD patients [39–41].

The vast majority of epigenetic studies of *5-HTTLPR* did not consider *SL* genotype individually, which makes it hard to determine the real (in)consistency of data [8]. Similar to Iga et al., we found highermethylation at CpG 21 in depressed older individuals with the LL genotype, although this was only nominally significant in our study. Interestingly, previous findings also indicate that age can influence the association between *5-HTTLPR* and mental health [8].

Given the larger sample size, our findings further strengthen the evidence that *5-HTTLPR* plays a role in modifying the association of methylation with depression. We also found significant modifying effects from the triallelic *5-HTTLPR/rs25531* polymorphism. Whilst *5-HTTLPR/rs25531* modification effects have not been studied in relation to the CpG island, a recent study found that carriers of the S′ allele had lowermethylation at a neighboring Alu retrotransposon (AluJb) in association with stress [42], which is in a similar direction of effect found in our study.

This is also the first study to examine the potential modifying effects of other polymorphisms throughout the *SLC6A4* gene on the depression-methylation association. Here, we report modifying effects from *rs140700, rs4251417, rs6354*, but these associations did not remain significant following multivariate adjustment. This may be because of the relatively small sample size with these genotypes, in particular because there were too few minor homozygotes to include in the analysis, with the comparison instead been only between major homozygotes and heterozygotes. Therefore, a larger sample size able to capture the minor homozygote is needed to further investigate these associations. Overall, the effects of genotype and methylation may combine to exert another layer of regulation in modifying risk of depression. Further investigation is needed to examine the underlying mechanisms and its function in influencing depression risk.

Our study of 302 participants is larger than all previous studies investigating *SLC6A4* methylation and genetic variation in depression. However only subtle differences in DNA methylation levels have been observed in peripheral tissues in association with psychiatric disorders, suggesting that an even larger sample size may be required to fully reveal true associations. We were able to consider several polymorphisms throughout the gene in combination with promoter methylation in our study, and adjust for a wide variety of confounding factors including sex, age and antidepressant use. Our participants were from the general population, thus making our findings more generalisable, as opposed to studies focused only on depressed hospital patients. Late-life depression covers a range of mild to severe depressive symptoms [43], so the CES-D scale to assess depressive symptoms, together with the DSM-IV classification of MDD (American Psychiatric Association, 1994), has helped ensure non-depressed individuals in this study did not have significant levels of sub-clinical symptomatology. Whilst our results may be generalisable to the older population, it may not be the case for earlier onset depression and non-Caucasian populations. Contributors to the etiology of depression may vary by age of onset, with late-life depression more frequently comorbid with physical and psychiatric conditions such as cardiovascular disease and stroke [44]. Depressive symptoms are also more frequent amongst the oldest old, which may be explained by factors associated with aging, such as higher proportion of women and increased physical impairment [44]. This is consistent with our finding that depressed individuals are significantly more likely to be female, older and functionally impaired (Table 1). DNA methylation patterns have been reported to vary with age, sex and ethnicity [45, 46]. Specifically, global hypomethylation has been reported in females [46] and with increasing age [45]. Hence, decreases in *SLC6A4* methylation, as observed in this study, may be specifically associated with late-life depression.

Other limitations to our study are the cellular heterogeneity of blood, the cross-sectional design of this study and potential residual confounding from factors for which information was not available or collected. Our study focused on white blood cells, which contain a heterogenous assortment of cell types. Given the cell-type-specific nature of methylation, variation in cellular composition may lead to distinct methylation profiles between cell types, potentially confounding methylation-related analyses [47]. This may account for the lack of associations observed between depression and *SLC6A4* methylation in our study. Cellular heterogeneity cannot be controlled for in a candidate gene study such as ours. Epigenome-wide association studies, on the other hand, have the ability to computationally adjust for cellular heterogeneity [48], with the added advantage of being able to interrogate large proportions of the (epi)genome without an a priori hypothesis.

Finally, as an association study, we are unable to draw any conclusions about the functionality or causality of our findings. Using peripheral methylation to examine a brain-based disorder has its limitations, as methylation profiles can also be distinct across different tissues. A few studies have reported correlations between DNA methylation levels in blood and post-mortem brain tissue, although these are likely to be gene-specific [49, 50]. Changes in peripheral tissues, such as in inflammatory markers, have been observed in depression, therefore becoming increasingly recognised as a systemic disease [51, 52]. Further, both *SLC6A4* methylation and genetic variants have been correlated with brain structural changes, including the hippocampus and corpus callosum, in depressed individuals [12, 53–55]. As such, our study may provide support for *SLC6A4* methylation as a biomarker of depression, indicating that such a biomarker would need to consider *SLC6A4* methylation in combination with genetic variation.

## Conclusion

Our study of late-life depression did not find any strong evidence for an independent association between *SLC6A4* promoter methylation and depression, however this may be modified by underlying genetic variants in the region. Further investigation is needed to examine the mechanisms behind such interactions, and replication in larger, independent and longitudinal studies are needed to help confirm these findings.

## Additional file

Additional file 1: **Table S1.** Individual CpG units assayed and analysed in this study. **Table S2.** Frequency of *SLC6A4* genotypes according to depression status in the study population. **Figure S1.** Flowchart of the quality control process following the generation of *SLC6A4* methylation data. **Figure S2.** Differences in DNA methylation according to depression status, stratified by *SLC6A4* genotypes. (DOCX 421 kb)

## Abbreviations

CES-D: Centre for Epidemiological Studies Depression Scale
DSM-IV: Diagnostic and Statistical Manual of Mental Disorders-IV
HWE: Hardy-Weinberg equilibrium
IQR: Interquartile range
MDD: Major depressive disorder
MINI: Mini International Neuropsychiatric Interview
mQTL: Methylation quantitative trait loci
SNP: Single nucleotide polymorphism
SSRI: Selective serotonin re-uptake inhibitor
VNTR: Variable number tandem repeat
