# Supplementary material for: Genotype-dependent associations between serotonin transporter gene (SLC6A4) DNA methylation and late-life depression
Source: BMC Psychiatry. 2018 Sep 4;18:282. doi: 10.1186/s12888-018-1850-4 (PMC6122720; doi:10.1186/s12888-018-1850-4)
Supplement: Supplementary file 1 — Table S1. Individual CpG units assayed and analysed in this study. Table S2. Frequency of SLC6A4 genotypes according to depression status in the study population. Figure S1. Flowchart of the quality control process following the generation of SLC6A4 methylation data. Figure S2. Differences in DNA methylation according to depression status, stratified by SLC6A4 genotypes. (DOCX 421 kb) [file 12888_2018_1850_MOESM1_ESM.docx]

Supplementary Material

**Genotype-dependent associations between serotonin transporter gene (*SLC6A4*) DNA methylation and late-life depression**

Dilys Lam, BSc (Hons); Marie-Laure Ancelin, PhD; Karen Ritchie, PhD; Rosanne Freak-Poli, PhD; Richard Saffery, PhD; Joanne Ryan, PhD

**Table of Contents**

**Table S1.** Individual CpG units assayed and analysed in this study

**Table S2.** Frequency of *SLC6A4* genotypes according to depression status in the study population

**Figure S1.** Flowchart of the quality control process following the generation of *SLC6A4* methylation data.

**Figure S2.** Differences in DNA methylation according to depression status, stratified by *SLC6A4* genotypes

**Table S1**. Individual CpG units assayed and analysed in this study

| CpG unit | Position (chr17) | Included | Reason for exclusion | *n* = | Mean methylation (%) | SD |
| --- | --- | --- | --- | --- | --- | --- |
| 1.2 | 30235765  30235768 | Yes |  | 294 | 3.08 | 1.07 |
| 3 | 30235795 | Yes |  | 299 | 2.81 | 1.43 |
| 4 | 30235808 | No | Same mass as CpG 24, thus identical results | NA | NA | NA |
| 5.6.7.8.9.10.11 | 30235829  30235831  30235835  30235837  30235743  30235845  30235851 | No | No data | NA | NA | NA |
| 12.13 | 30235866  30235870 | Yes |  | 301 | 5.85 | 1.62 |
| 14.15 | 30235884  30235886 | Yes |  | 297 | 2.03 | 1.14 |
| 16.17.18.19.20 | 30235891  30235896  30235903  30235905  30235909 | Yes |  | 256 | 6.95 | 2.30 |
| 21 | 30235939 | Yes |  | 282 | 4.08 | 1.85 |
| 22.23 | 30235952  30235956 | Yes |  | 298 | 2.57 | 1.03 |
| 24 | 30235968 | No | Same mass as CpG 4, thus identical results | NA | NA | NA |
| 25.26 | 30235973  30235975 | Yes |  | 242 | 27.4 | 5.98 |
| 27 | 30235984 | Yes |  | 295 | 1.14 | 0.91 |
| 28 | 30235998 | Yes |  | 290 | 2.72 | 1.39 |
| 29 | 30236002 | No | No data | NA | NA | NA |
| 30 | 30236036 | Yes |  | 290 | 2.72 | 1.39 |

**Table S2.** Frequency of *SLC6A4* genotypes according to depression status in the study population

| Genetic variant and genotype | No Depression (%) | Depression (%) | *p-*value^a^ |
| --- | --- | --- | --- |
| *rs140700* (n=151) |  |  |  |
| GG | 85.3 | 85.7 | 0.73 |
| GA | 12.9 | 14.3 |  |
| AA | 1.80 | 0.00 |  |
| *rs25528* (n=155) |  |  |  |
| AA | 73.1 | 66.7 | 0.70 |
| AC | 23.5 | 27.8 |  |
| CC | 3.40 | 5.50 |  |
| *rs4251417* (n=152) |  |  |  |
| GG | 80.3 | 77.1 | 0.49 |
| GA | 17.1 | 22.9 |  |
| AA | 2.60 | 0.00 |  |
| *rs6354* (n=154) |  |  |  |
| AA | 65.2 | 61.1 | 0.80 |
| AC | 31.4 | 33.3 |  |
| CC | 3.40 | 5.60 |  |
| *5-HTTLPR* (n=293) |  |  |  |
| LL | 27.9 | 38.2 | 0.023 |
| SL | 53.4 | 36.0 |  |
| SS | 18.7 | 25.8 |  |
| *5-HTTLPR/rs25531* (n=220) |  |  |  |
| S’S’ | 26.9 | 34.4 | 0.15 |
| S’L’ | 51.9 | 37.5 |  |
| L’L’ | 21.2 | 28.1 |  |
| *rs25531* (n=297) |  |  |  |
| AA | 87.3 | 88.0 | 0.35 |
| AG | 12.2 | 9.79 |  |
| GG | 0.50 | 2.12 |  |

^a^ Chi-squared tests were used to calculate *p*-value


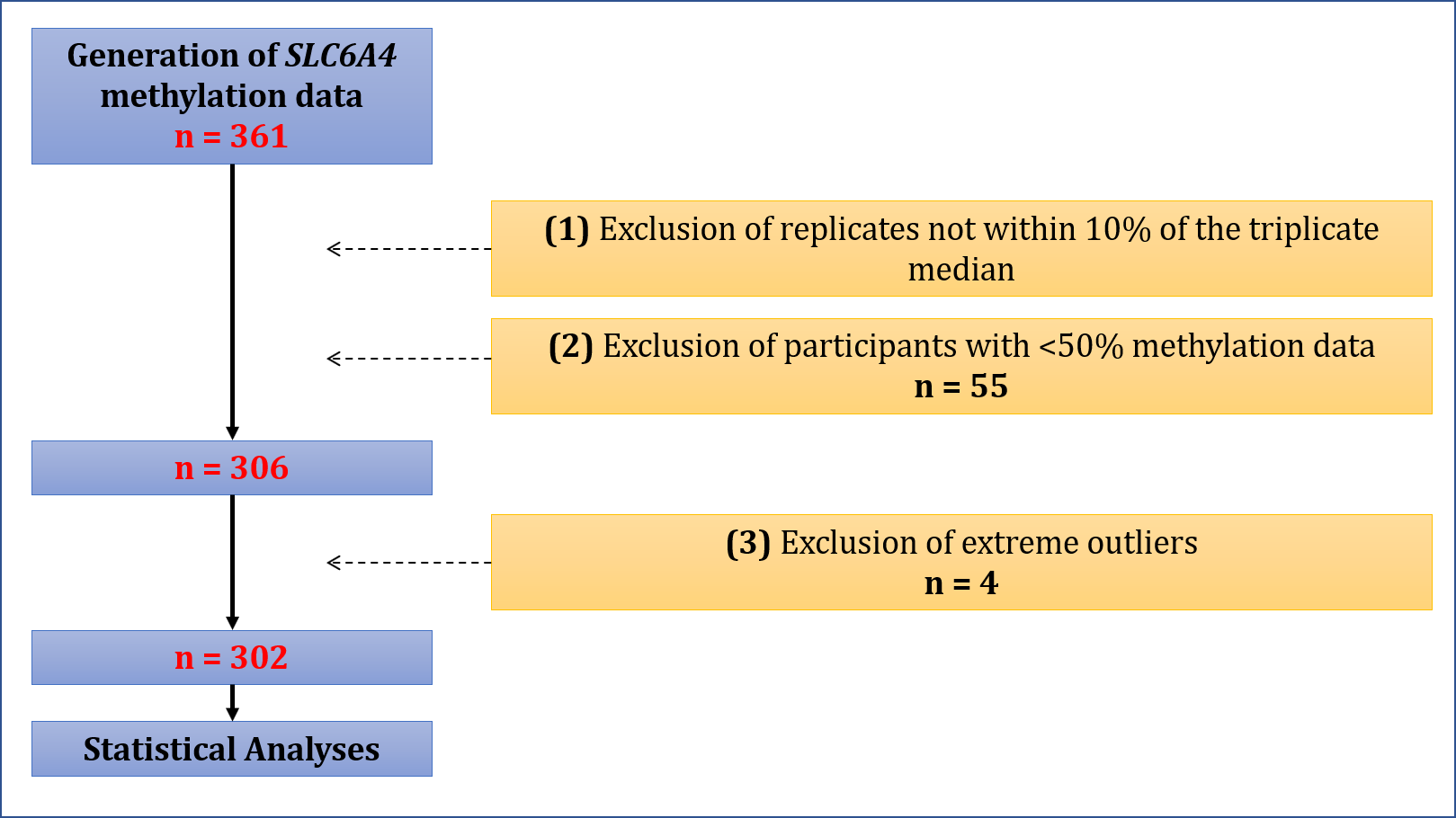


**Figure S1.** Flowchart of the quality control process following the generation of *SLC6A4* methylation data. The number of samples following each quality control step is shown on the left hand panel (blue), with the right hand panel (orange) indicating the quality control steps undertaken and how many samples were excluded in each step.

**
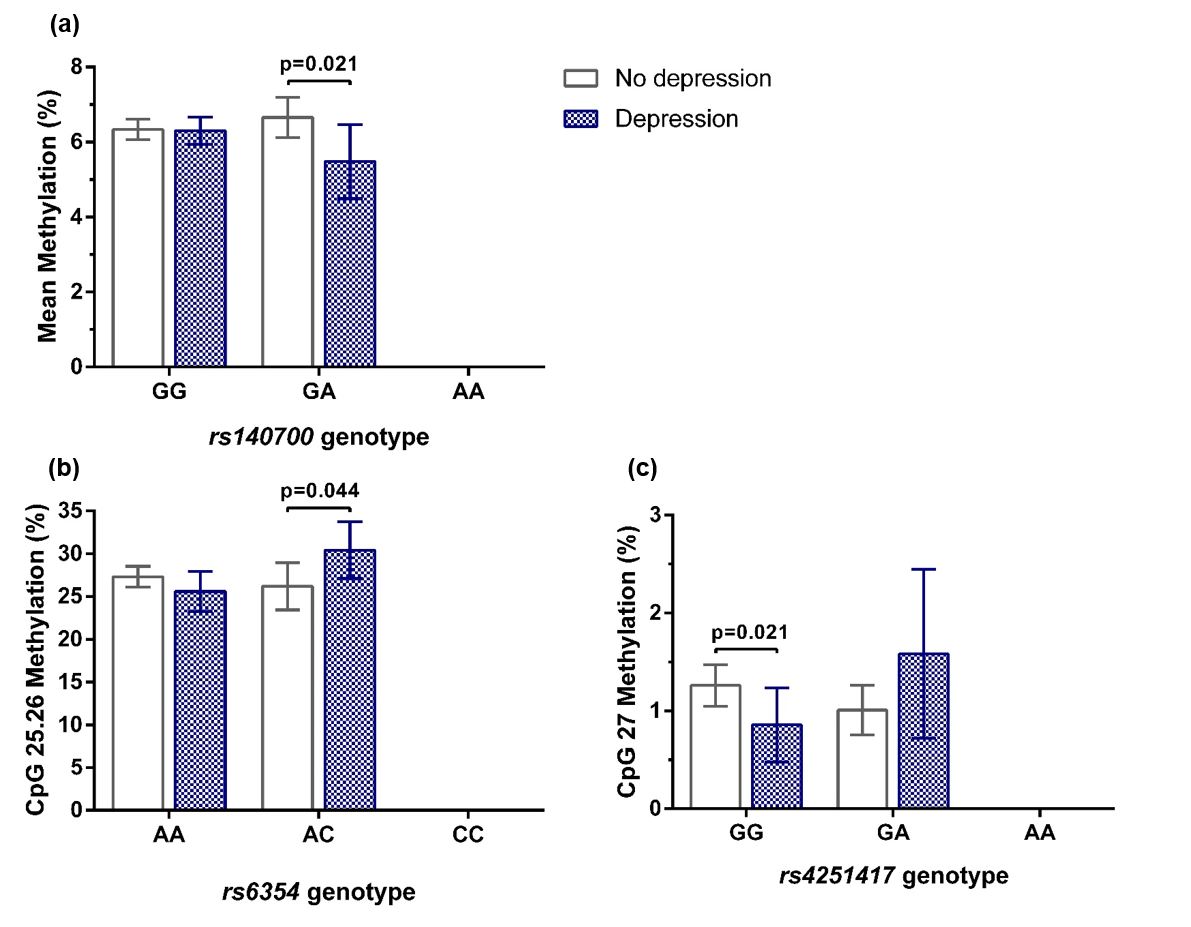
Figure S2.** Differences in DNA methylation according to depression status, stratified by *SLC6A4* genotypes: (a) Mean Methylation (*rs140700,* n = 104), (b) CpG 25.26 (*rs6354,* n = 126), (c) CpG 27 (*rs4251417,* n = 143). Data presented as the average methylation ± 95% CI. T-tests were used to calculate *p*-values, with significant associations at p < 0.05 indicated in the figure.
